# Unlocking lung regeneration: insights into progenitor cell dynamics and metabolic control

**DOI:** 10.1186/s13619-024-00212-y

**Published:** 2024-12-16

**Authors:** Jiaying Yang, Yawen Li, Ying Huang, Huaiyong Chen, Pengfei Sui

**Affiliations:** 1 State Key Laboratory of Cell Biology, Shanghai Institute of Biochemistry and Cell Biology, Center for Excellence in Molecular Cell Science, Chinese Academy of Sciences, University of Chinese Academy of Sciences, Shanghai, 200031 China; 2 Department of Basic Medicine, Tianjin University Haihe Hospital, Tianjin, 300350 China; 3 Tianjin Key Laboratory of Lung Regenerative Medicine, Tianjin, China; 4 Key Research Laboratory for Infectious Disease Prevention for State Administration of Traditional Chinese Medicine, Tianjin Institute of Respiratory Diseases, Tianjin, China; 5 Department of Basic Medicine, Haihe Clinical College of Tianjin Medical University, Tianjin, China

**Keywords:** Lung repair and regeneration, Lung progenitor cells, Cell metabolism, Cell fate decision

## Abstract

Regenerative responses are particularly important in the lungs, which are critical for gas exchange and frequently challenged by environmental insults. The lung progenitor cells play a central role in the lung regeneration response, and their dysfunction is associated with various lung diseases. Understanding the mechanisms regulating lung progenitor cell function is essential for developing new therapeutic approaches to promote lung regeneration. This review summarizes recent advancements in the field of lung regeneration, focusing on the metabolic control of lung progenitor cell function. We discuss cell lineage plasticity and cell–cell signaling under different physiological conditions. Additionally, we highlight the connection between progenitor cell dysfunction and lung diseases, emphasizing the need to develop new therapeutic strategies in regenerative medicine to improve lung regenerative capacity.

## Background

To maximize gas exchange capacity, the lung has evolved elegant structures that increase its surface area. As the lung epithelium is constantly exposed to the external environment, it faces various stresses, including chemical, mechanical, biological, immunological, and xenobiotic factors. This highlights the importance of regenerative responses mediated by progenitor cells for lung integrity.

Several types of progenitor cells have been identified within the lung epithelium, which distribute across different anatomical regions. These cells respond to injury signals to repair damaged epithelium, restoring tissue integrity and function. Key progenitor cells include basal cells in the trachea (Rock et al. 2009), club cells in the airway (Rawlins et al. 2009), myoepithelial cells in the submucosal glands (SMGs) (Lynch et al. 2018; Tata et al. 2018), bronchoalveolar stem cells (BASCs), and p63^+^ progenitor cells in the distal airway (Kim et al. 2005; Vaughan et al. 2015; Xi et al. 2017; Zuo et al. 2015), and alveolar type 2 (AT2) cells in the alveoli (Barkauskas et al. 2013).

Advancements in lineage tracing, transcriptomics, and metabolomics technologies have led to a paradigm shift in our understanding of cell plasticity and fate regulation of lung progenitor cells, moving from a simplistic, linear view of lung progenitor cell differentiation to a more complex, dynamic model. This paradigm shift provides new insights into lung development, homeostasis, and disease, potentially leading to novel therapeutic strategies for lung diseases. In this work, we review recent advancements in cellular complexity, lineage plasticity, and the regulatory mechanisms governing lung progenitor cell function, with a focus on the metabolic control of lung progenitor cell fate decisions. The emerging area of researches increasingly recognized for its importance in regulating progenitor cell maintenance and regenerative activity. Furthermore, we summarize existing evidence linking lung progenitor cell dysfunction to human diseases. Understanding these processes will inform future research directions and the development of clinical applications for respiratory diseases.

## Diverse lung progenitor cell lineages

**Fig. 1:**
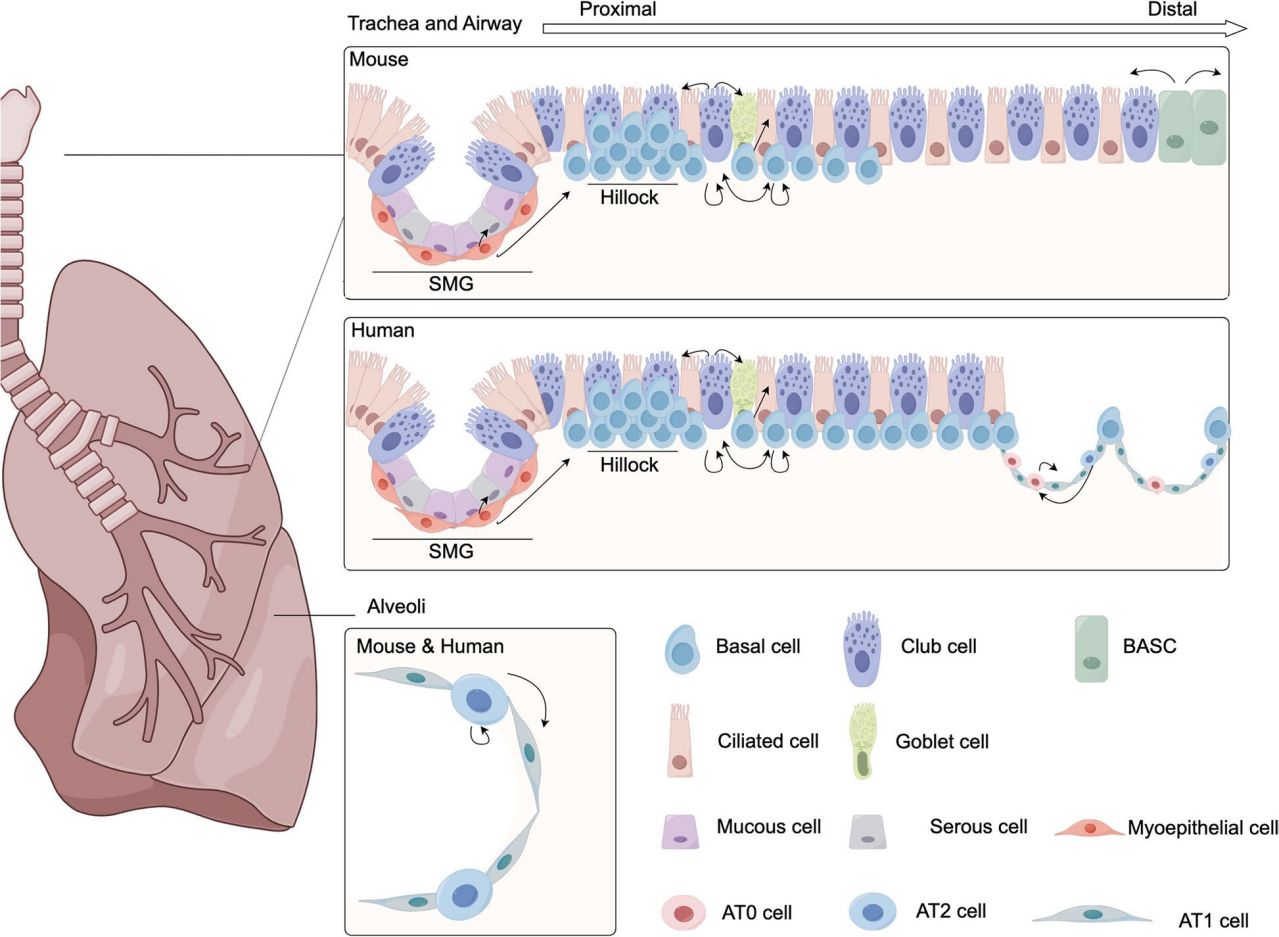
Structure of the lung epithelium and organization of major epithelial cell types. A schematic of the lung, highlighting the anatomic regions within the respiratory system. Proximal airways are lined with pseudostratified columnar epithelium, which contains basal cells and several types of luminal cells. Basal cells are the dominant stem cells that can give rise to almost all epithelial cell types and form hillocks. Club cells are recognized as progenitor cells capable of differentiating into both airway and alveolar cell types. Submucosal glands (SMGs) myoepithelial cells function as reserve stem cells capable of regenerating tracheal epithelium. Bronchoalveolar stem cells (BASCs) are transcriptionally different from club cells and AT2 cells. They process the capability to differentiate into distal airway club cells and ciliated cells following naphthalene-induced injury or differentiate into AT2 cells. Alveoli, the gas exchange units of the lung, are mainly comprised of AT1 and AT2 cells. AT2 cells function as progenitor cells in the alveoli, with the ability to proliferate and differentiate into AT1 cells

From proximal to distal, the lung is comprised of three major structures: proximal airways (from the trachea to the main bronchi), distal airways (from the branching of bronchi to the alveoli, including the bronchioles and the terminal bronchioles), and alveoli. Each of these structures contains distinct epithelial types and varying populations of progenitor cells. The tracheal epithelium is lined with pseudostratified columnar epithelium, primarily maintained by basal cells. Additionally, myoepithelial cells in the SMG can function as reserve stem cells to regenerate airways after injury. In both the proximal and distal airways, club cells serve as the progenitor cells, capable of differentiating into both airway and alveolar cell types. In the alveoli, AT2 cells are the primary progenitor cell type for homeostatic turnover and regeneration. However, during severe damage, cells in the distal airway, such as club cells, BASCs, and intrapulmonary p63^+^ cells, can migrate into the alveoli to repair injured alveoli (Fig. 1). In this section, we will review the diversity and lineage plasticity of these lung progenitor cell populations.

### Tracheal and airway epithelial progenitor cells

#### Basal cells

Basal cells are characterized by the expression of P63 (Transformation related protein 63), KRT5 (Keratin 5), NGFR (Nerve growth factor receptor), and KRT14 (Keratin 14) (Lee et al. 2014a, b, c; Van Keymeulen et al. 2011). Basal cells are multipotent stem cells capable of generating all cell types of the tracheal airway epithelium, including ciliated cells, club cells, tuft cells, goblet cells, and neuroendocrine cells (Hong et al. 2004; Montoro et al. 2018). Interestingly, in addition to serving as stem cells, basal cells also function as niches that maintain luminal cell identity and act as sensors for environmental cues. (Tata et al. 2013). Though with similar differentiation potential, the distribution of basal cells is more broadly in humans than mice. In mice, basal cells reside in the trachea and proximal main stem bronchi, whereas in human they extend into the intrapulmonary airways (Basil et al. 2020).

#### Airway hillock basal cells

Airway hillocks are flat-topped structures recently identified within the proximal airway. They are composed of a stratified upper layer of scale-like squamous cells on top of an underlying layer of rapidly expanding basal cells (Lin et al. 2024a, b)*.* Hillock basal cells are labeled by KRT13 (Keratin 13), KRT6A (Keratin 6A), and DSG3 (Desmoglein 3) (Lin et al. 2024a, b; Montoro et al. 2018). Given the stratified hillock structure confer them with broadly resistant to a range of injuries, working as injury-resistant reservoirs. Following injury, the hillock basal stem cells can undergo massive expansion to resurface denuded airways and eventually regenerate normal airway epithelium. Under homeostasis, hillock basal cells also exhibit a much higher turnover rate compared to the largely quiescent classic airway basal progenitors, which continually replenish the overlying squamous barrier cells (Lin et al. 2024a, b). Stimulation of basal cells with retinoic acid signaling promotes the formation of squamous barrier structures in vitro (Lin et al. 2024a, b). Furthermore, the hillocks are one origin of ‘squamous metaplasia’, which has been considered as a precursor of lung cancer (Lin et al. 2024a, b).

#### Club cells

Club cells are the major type of secretory cells in the airway epithelium, which also function as progenitor cells and can differentiate into both airway and alveolar cell types. They secrete various substances, including glycosaminoglycans, proteins such as lysozymes, and club cell secretory protein (CCSP). These secretions contribute to the formation of a protective lining in the airways. The most used markers for club cells are SCGB1A1 (Secretoglobin family 1A member 1), also known as CCSP (Club cell secretory protein), and SCGB3A2 (Secretoglobin family 3A member 2) (Sun et al. 2022). Club cells exhibit varying differentiation potentials across different lung regions, which are regulated by surrounding microenvironment. In the trachea, when basal cells are depleted, club cells can dedifferentiate into basal cells to regenerate the tracheal epithelium (Tata et al. 2013). In the airway, during homeostasis and injury repair, club cells maintain the integrity of the intrapulmonary epithelium via self-renewal and differentiation into ciliated cells and goblet cells (Rawlins et al. 2009; Rock et al. 2011). In the distal airway, following severe alveolar damage, club cells can also migrate from the distal airway into the alveoli and differentiate into AT2 cells (Liu et al. 2024; Zheng et al. 2012, 2017). During this process, club cells may also give rise to p63^+^ progenitor cells, which further differentiate into AT2 and AT1 cells (Lv et al. 2024).

Additionally, a subset of club cells expressing UPK3A (Uroplakin 3A) in mice, termed variant-club cells, exhibits resistance to naphthalene-induced damage due to low expression of cytochrome CYP2F2 (Cytochrome P450, family 2, subfamily f, polypeptide 2). These variant-club cells can generate both club cells and ciliated cells during development and in response to injury (Giangreco et al. 2009; Guha et al. 2017).

#### Myoepithelial cells

Submucosal glands (SMGs) are grape-like tubular structures embedded within the mesenchyme beneath the surface airway epithelium of all cartilaginous airways in humans and the proximal trachea of mice (Liu et al. 2004; Widdicombe et al. 2015). The markers of SMG myoepithelial cells are KRT14 and MYH11 (Myosin heavy chain 11) (Anderson et al. 2017), and these cells have been shown to function as reserved progenitor cells for tracheal epithelium regeneration. Recent studies suggest that myoepithelial cells in SMGs can differentiate into both SMGs and airway epithelial cell types when tracheal epithelium is severely damaged (Lynch et al. 2018; Tata et al. 2018).

SMGs in mice are only found in the most proximal part of the airway, whereas in human and other larger mammals, SMGs are found throughout the cartilaginous airways (Liu et al. 2008), which indicates a more important role of SMGs in lung regeneration than their role in mice.

#### Bronchoalveolar stem cells (BASCs)

Bronchoalveolar stem cells (BASCs) are rare distal progenitor cell population characterized by the co-expression of two key markers: SCGB1A1 (associated with club cells) and SFTPC (associated with AT2 cells) (Liu et al. 2019; Salwig et al. 2019). This population was first identified in bronchioalveolar duct junction in mice in 2005 (Kim et al. 2005). BASCs are transcriptionally different from club cells and AT2 cells but process the capability to differentiate into distal airway club cells and ciliated cells following naphthalene-induced injury or differentiate into AT2 cells after bleomycin or influenza injury (Liu et al. 2024; Liu et al. 2020, 2019; Salwig et al. 2019). While BASCs have been well-characterized in mice, their direct equivalent in human lungs has not been definitively identified (Sun et al. 2022). Further research is needed to identify and characterize the equivalent progenitor cell population in the human lung.

#### p63^+^ progenitor cells

P63^+^ progenitor cells represent a rare population of basal-like cells located in the distal airway. Following severe influenza injury, such as those caused by H1N1, p63^+^ progenitor cells can give rise to KRT5^+^ cells to repair the injured alveoli (Hers et al. 1958; Kumar et al. 2011; Loosli et al. 1975). (Lee et al. 2014a, b, c; Vaughan et al. 2015; Xi et al. 2017; Yang et al. 2018; Zuo et al. 2015). Interestingly, these infection-induced KRT5^+^ progenitor cells do not differentiate into alveolar cell types but give rise to tuft cells and goblet cells in the injured alveoli (Fernanda de Mello Costa et al. 2020; Weiner et al. 2022; Yang et al. 2018).

The expansion of p63^+^ progenitor cells in alveoli has also been observed in bleomycin injured lungs. Compared to flu injured lungs, these p63^+^ progenitor cells have a higher potential to produce AT2 cells, with approximately one-third of KRT5-lineage-labeled cells expressing AT2 markers (Vaughan et al. 2015; Weiner et al. 2022). Furthermore, the latest genetic-lineage tracing study using dual recombinases demonstrated that these p63^+^ progenitor cells are derived from airway secretory cells (Lv et al. 2024).

Although p63^+^ progenitor cells are induced following both influenza infection and bleomycin treatment, their differentiation potential is completely different. Future studies that carefully compare the cell plasticity of p63^+^ progenitor cells induced by these different injury models will provide further insights into their lineage commitment and functional outcomes.

### Alveolar progenitor cells

#### Alveolar type 2 (AT2) cells

AT2 cells are characterized by the expression of surfactant proteins, particularly SFTPC (Surfactant Protein C), which is one of the most used markers for AT2 cells. Additionally, the lamellar body-associated proteins LAMP3 (Lysosomal associated membrane protein 3) and ABCA3 (ATP binding cassette subfamily A member 3) are also markers for AT2 cells (Barkauskas et al. 2013). AT2 cells proliferate and differentiate into alveolar type 1 (AT1) cells. The turnover of AT2 cells is relatively slow under homeostatic conditions. However, following alveolar damage, they rapidly differentiate into AT1 cells (Barkauskas et al. 2013; Desai et al. 2014). Recent studies suggest that during differentiation, AT2 cells first enter a primed state, then become transitional AT2 cells, and ultimately differentiate into mature AT1 cells. The transitional state is marked by the expression of KRT8 (Keratin 8) and CLDN4 (Claudin 4) (Choi et al. 2020; Kobayashi et al. 2020; Strunz et al. 2020). Interestingly, in humans, a new cell type called respiratory bronchioles-specific alveolar type 0 (AT0) cells was recently identified. These cells are conserved in primates and emerge during human lung development. Using a non-human primate lung injury model, along with human organoids and tissue specimens, it has been shown that AT2 cells in regenerating lungs transiently acquire an AT0 state before differentiating into AT1 cells (Kadur Lakshminarasimha Murthy et al. 2022).

One mouse lung contains more than one million AT2 cells. Identifying the subpopulations of AT2 cells with more regenerative potential is currently under intensive investigation. Several subpopulations, such as Axin2^+^ cells (Wnt-responsive alveolar epithelial progenitors, AEPs) (Nabhan et al. 2018; Zacharias et al. 2018), CD44^+^ AT2 cells (Chen et al. 2017), and Pdl1^+^ AT2 cells (Ahmadvand et al. 2021) have been identified. However, the anatomical location and specific niche of these subpopulation remain unclear. Whether these cells are intrinsically different from other AT2 cells, or simply transiently upregulate specific genes in response to environmental cues is still debated.

Notably, under normal culture conditions, human AT2 cells do not readily differentiate into AT1 cells, which leads to a lack of models for studying human AT2-to-AT1 cell differentiation. Recent studies generated human AT1 and AT2 cells from induced pluripotent stem cells (iPSCs). Using this model, they found that inhibiting Wnt signaling or activating nuclear YAP (Yes-associated protein) expression may facilitate human AT2 to AT1 differentiation in vitro (Abdelwahab et al. 2019; Burgess et al. 2024; Kanagaki et al. 2021; Ohnishi et al. 2024). However, the resulted AT1 cells still express much lower level of mature AT1 cell markers, such as *Ager* (Advanced glycosylation end-product specific receptor) and *Pdpn* (Podoplanin) compared to AT1 cells in vivo, calling for a more comprehensive understanding of the potential mechanisms controlling human AT2 cell differentiation.

## Signaling in the progenitor cell niche: regulating cell fate, function and plasticity

**Fig. 2:**
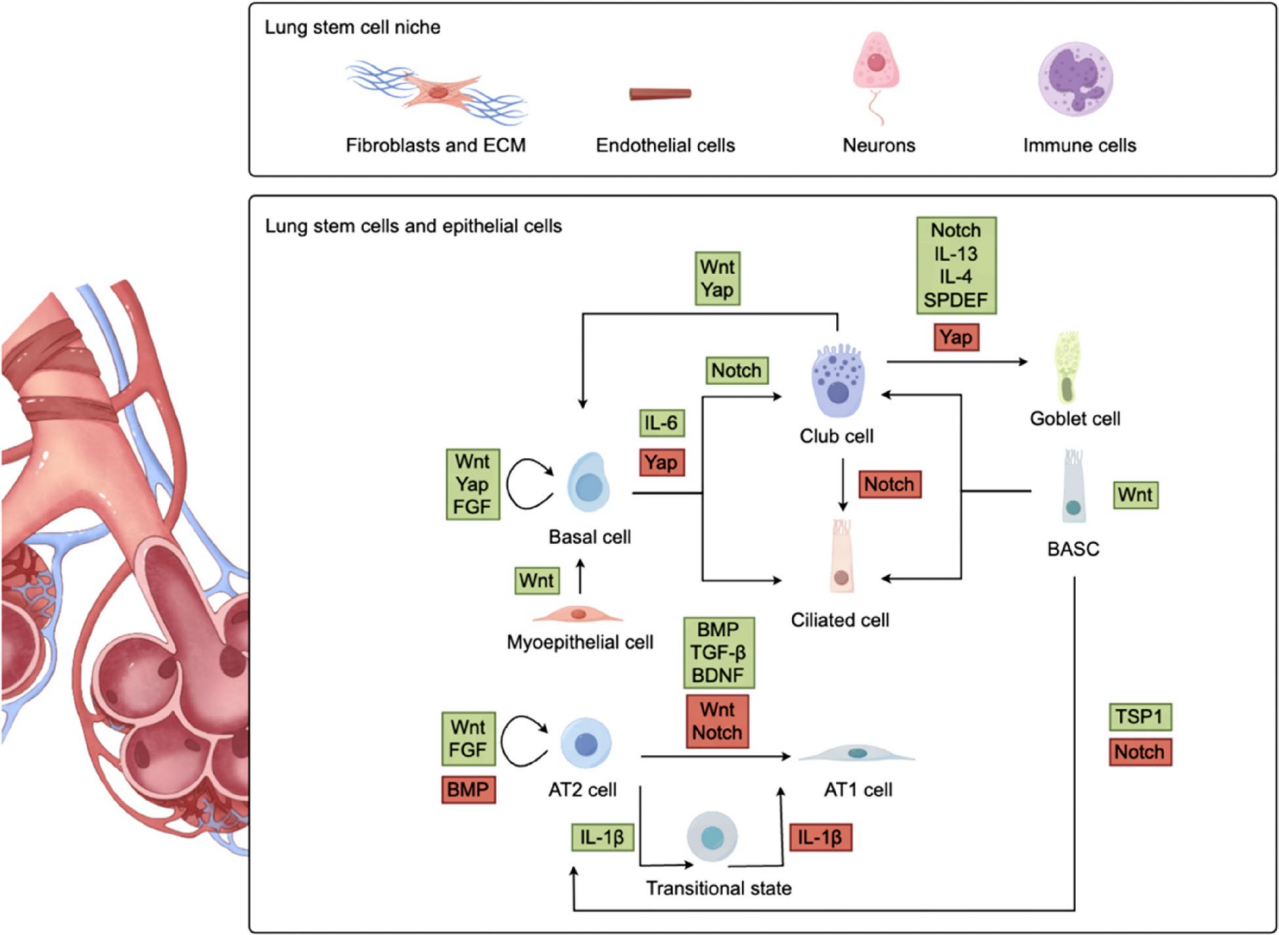
An overview of the main signaling pathways regulating lung progenitor fate decision. Signaling pathways that regulate the function of lung progenitor cells. The self-renewal of basal cells is regulated by Wnt, Yap, and FGF signaling. Notch and IL-6 signaling are required for basal cell differentiation. Additionally, Notch signaling modulates the balance between ciliated and secretory cells. The transition of myoepithelial cells in SMGs is regulated by Wnt signaling. The differentiation of club cells into goblet cells is mediated by Notch, Yap, and some type-2 cytokines. Under specific conditions, Wnt and Yap signaling drive club cell differentiation into basal cells. Notch signaling inhibits BASCs differentiation, while Wnt signaling increases their number. Thrombospondin-1 (TSP1) expression in lung endothelial cells guides BASCs toward alveolar-specific differentiation. AT2 cell identity is maintained by the Wnt and FGF signaling. Reduced BMP signaling in AT2 cells promotes their self-renewal. BMP, TGF-β, and BDNF signaling support AT2 cell differentiation, while Wnt and Notch signaling inhibit it. Injury-induced IL-1β modulates AT2 cell differentiation, leading to impaired alveolar regeneration. The green box represents positive regulation whereas the red box represents negative regulation

The fate of lung progenitor cells is dynamically regulated by signals from their microenvironment (Fig. 2). The lung progenitor cell niche is composed of various cell types, including fibroblasts, endothelial cells (ECs), neurons, immune cells, as well as the surrounding microenvironment, which includes extracellular matrices (ECMs) and signaling factors. Specifically, fibroblasts produce ECMs, which provide structural support, as well as signaling cues that are required for progenitor cell function (Chen et al. 2012; Gomes et al. 2021; Plantier et al. 2007). ECs contribute to alveolar development by forming basement membranes that support myofibroblasts (Watanabe-Takano et al. 2024). Growth factors released by ECs stimulate epithelial cell growth, aiding in primary septa formation during lung development and regeneration (Ding et al. 2011; Yamamoto et al. 2007). Pulmonary neuroendocrine cells (PNECs) influence lung progenitor cell activity, potentially affecting cell function through neuropeptide regulation like GABA (Barrios et al. 2019; Branchfield et al. 2016). In addition, immune cell-derived signals, such as cysteinyl leukotrienes, IL-1β, IL-13, and IL-6 also regulate lung progenitor cell differentiation (Barnes et al. 2023; Glisinski et al. 2020).

### Basal cells

The activation and differentiation of basal cells are regulated by several signaling pathways. Wnt/β-catenin signaling is initiated by the binding of Wnt ligands to Frizzled receptors and co-receptors, such as LRP5/6, leading to the stabilization of β-catenin and its subsequent translocation into the nucleus to regulate target gene expression (Liu et al. 2022). Basal cells exhibit elevated Wnt/β-catenin signaling activity during regeneration. Inhibition of this signaling leads to decreased cell proliferation and impair epithelial repair (Giangreco et al. 2012). Forced activation of Wnt/β-catenin signaling in basal cells using K14-Δβ-catenin ER mice restricts ciliated cell differentiation (Giangreco et al. 2012). In contrast, air–liquid interface culture experiment indicated that persistent β-catenin signaling in basal cells promotes ciliated cell differentiation at the expense of club-like cells (Brechbuhl et al. 2011). A recent study identified that the PDGFRα^+^ lineage cells within the intercartilaginous zone (ICZ) niche promotes basal cell expansion via secreting Wnt ligands. Interestingly, basal cells themselves also serve as a source of Wnt signals. Basal cell-derived Wnt signal is required for basal cell proliferation and regulate the differentiation of ciliated cells (Aros et al. 2020). Immune signals, such as IL-6, are also mediators for airway repair, which stimulates basal cells to differentiate into ciliated and secretory cells following naphthalene induced injury (Tadokoro et al. 2014).

The YAP signaling pathway which regulates cell–cell contact, is critical for basal cell fate regulation. In the adult lung, inactivation of YAP leads to unchecked basal cell differentiation, while its overexpression enhances progenitor cell self-renewal and inhibits differentiation, resulting in hyperplasia and epithelium stratification (Zhao et al. 2014). Following injury, increased nuclear YAP in proliferating basal cells enhance WNT7a secretion, which upregulates *Fgf10* expression in stromal niche to further promote their expansion via *Fgf10-Fgfr2b* signaling (Volckaert et al. 2017; Wei et al. 2023). The activation of YAP signaling is a complex process. Multiple stimuli can induce nuclear Yap. For instance, YAP can be regulated by Hippo signaling pathway components such as MST1/2 and LATS1/2, which phosphorylate YAP and regulate its subcellular localization and activity. Additionally, YAP activity is also modulated by other pathways, such as GPCR signaling, S1P signaling, and TGF-β signaling, which indirectly influence its function (Tang et al. 2022). Therefore, identifying the context specific cue for YAP activation is worth further investigation.

Notch signaling is a highly conserved pathway crucial for short-range cellular communication, which signals via direct cell–cell contact (Kiyokawa et al. 2020). In basal cells, Notch signaling is required for cell differentiation rather than self-renewal (Rock et al. 2011). Sustained activation of Notch in basal cells promotes their differentiation towards luminal cell fates, particularly secretory lineages (Rock et al. 2011). Conversely, inhibition of *Notch* signaling in club cells results in ciliated cell differentiation (Tsao et al. 2009). These findings indicate that Notch signaling determines ciliated or non-ciliated cell fates in proximal progenitors. Basal cells also express Notch ligands, including *Dll1* and *Jagged2* (Rock et al. 2009). This basal cell-derived Notch signaling is required for maintaining club cell homeostasis (Pardo-Saganta et al. 2015).

### Club cells

Club cells can differentiate towards the airway epithelial cell types and alveolar cell types. Wnt/β-catenin signaling promotes the differentiation of club cells into alveolar cell types rather than airway cell types (Hu et al. 2020; Lee et al. 2017; Mucenski et al. 2005). Notch signaling as mentioned previously is also critical for club cell fate decision (Morimoto et al. 2010; Tsao et al. 2009; Zhang et al. 2013). Activation of Notch signaling promotes club cell differentiation, while inhibition of Notch leads to a decrease in club cells and an increase in ciliated cells (Rock et al. 2011). Specifically, Jagged1-Notch2 signaling determines the cell fate decision between club and ciliated cells. *Notch1* activation also contributes to club cells specification, though it plays an auxiliary role compared to *Notch2* (Kiyokawa et al. 2020). Consistently, high Notch activity promotes the differentiation of club cell into goblet cells (Kim et al. 2019; Kiyokawa et al. 2020). Increased Notch signaling suppresses *p63* and *Gmnc*, while activating *Spdef*, which regulates a transcriptional network essential for airway goblet cell differentiation and mucin synthesis (Chen et al. 2009; Rajavelu et al. 2015). Acute Jagged blockade, such as using specific antibodies, rapidly converts club cells into ciliated cells (Lafkas et al. 2015). Importantly, Jagged nebulization using antibodies is able to reverse goblet cell metaplasia in a preclinical asthma model, showing therapeutic potential (Lafkas et al. 2015). Notch signaling is also regulated by inflammatory signals. Following bleomycin damage, IL-1β downregulates *Jag1* and *Jag2* expression in ciliated cells, resulting in reduced Notch activity in secretory cells thereby promoting their differentiation into AT2 cells (Choi et al. 2021).

Yap/Taz in club cells is required to prevent goblet cell metaplasia. Inhibition of Yap/Taz activity leads to an increase in goblet cell numbers and mucus production (Hicks-Berthet et al. 2021). In addition, forced activation of Yap in club cells can lead to partial reprogramming towards a stem cell-like state (Zhao et al. 2014). Moreover, type-2 cytokines, such as interleukin IL-4 and IL-13 also promote goblet cell differentiation (Seibold 2018; Zhu et al. 1999). Elevated levels of these cytokines are associated with lung diseases such as asthma and chronic obstructive pulmonary disease (COPD), where goblet cell hyperplasia and mucus overproduction are common pathological features (Balkrishna et al. 2024; Boomer et al. 2024; Nakagome et al. 2024).

### Myoepithelial cells

Previous studies have demonstrated that the activation of myoepithelial cells in SMGs is mainly regulated by Wnt-regulated transcriptional factors (Lynch et al.,^2018^; Tata et al. 2018). This activation induces *Lef-1* expression in cultured myoepithelial cells within SMGs, facilitating their transition into a basal cell phenotype. Intriguingly, conditional activation of *Lef-1* in myoepithelial cells in vivo resulted in self-limited airway regeneration response in a dose-dependent manner, even in the absence of injury (Lynch et al. 2018; Tata et al. 2018). Our understanding of the mechanisms regulating myoepithelial cell fate decisions is still limited.

### Bronchoalveolar stem cells (BASCs)

BASCs are cells found at the bronchioalveolar junction. Both club cells and BASCs exhibit downregulated Notch signaling activity when differentiating towards AT2 cells. Interestingly, while Notch signaling inhibits the differentiation of club cells, its suppression in BASCs enhance their ability to differentiate into AT2 cells (Liu et al. 2024). During lung epithelial repair, canonical Wnt signaling is activated in the niche containing BASCs. Forced activation of Wnt signaling leads to a dramatic increase in BASC numbers (Zhang et al. 2008). It has also been found that BMP4-Bmpr1a signaling induces thrombospondin-1 expression in lung endothelial cells, guiding BASCs toward alveolar-specific differentiation (Lee et al. 2014a, b, c), highlighting the role of endothelial cells in regulating lung progenitor cells.

###  P63^+^ progenitor cells

The activation of p63^+^ progenitor cells in mouse influenza model is regulated by Hif, Notch, YAP, and immune-related signaling pathways (Vaughan et al. 2015; Xi et al. 2017). Deletion of HIF1α in Sox2-CreERT2 mice reduces KRT5^+^ pod expansion. (Xi et al. 2017). Inhibition of Notch also leads to a reduction in KRT5^+^ cells formation (Vaughan et al. 2015). Similarly, blocking YAP during viral infection prevent dysplastic KRT5^+^ cell formation, whereas inhibiting YAP in persistent KRT5^+^ cells led to their conversion into distal club cells (Lin et al. 2024a, b). Immune factors such as IL-22 and IFN-γ, which are robustly induced following infection, contribute to KRT5^+^ cell expansion (Beppu et al. 2023; Lin et al. 2024a, b).These data highlight the differences between the pathways that modulate p63^+^ cell fate. Future studies focused on developing new therapeutic strategy to target these cells will help lung function recover following viral infection.

### Alveolar type 2 (AT2) cells

The alveolar niche provides a short-range paracrine Wnt signaling that selects and maintains alveolar progenitor cell identity and proliferative capacity (Nabhan et al. 2018). Following injury, AT2 cells also initiate autocrine Wnt signaling to temporarily expand the progenitor cell pool (Nabhan et al. 2018). FGF signaling is another signaling pathway that regulates AT2 cells fate, which inhibits AT1 cell differentiation, and maintains AT2 cell identity. Inactivation of *Fgfr2* during in AT2 cells during early development reprograms them into AT1 cells fate, whereas in adult, it leads to apoptosis (Brownfield et al. 2022). Interestingly, findings from another study suggests that *Fgfr2* is not required for AT2 cell maintenance in adult lung (Liberti et al. 2021). The inefficient gene deletion efficiency and rapid recovery of AT2 cells from the remaining cells may explain why these two studies report contradictory observations (Brownfield et al. 2022).

AT2 cell fate is also regulated by BMP signaling, which mainly derives from alveolar niche. BMP signaling can act on both AT2 cells and PDGFRα^+^ stromal cells. Cell type-specific genetic manipulation experiment revealed that reduced BMP signaling in AT2 cells after PNX facilitates self-renewal, while increased BMP signaling promotes AT1 formation. Constitutive BMP signaling in PDGFRα^+^ cells diminish their AT2 support function (Chung et al. 2018). Similar to BMP, TGF-β signaling also promotes AT2 cell differentiation. Interestingly, TGF-β signaling is also required for maintaining AT1 cells fate. Disruption of TGF-β receptor 2 in AT1 cells during lung development reprograms these cells into AT2 cells (Callaway et al. 2024). The STAT3-BDNF-TrkB signaling pathway also regulates AT2 cell function, facilitating the repair of alveolar tissues by promoting the proliferation and differentiation of AT2 cells into AT1 cells. The receptor for BDNF, tropomyosin receptor kinase B (TrkB), is enriched on mesenchymal alveolar niche cells (MANCs), whereases BDNF is expressed in activated AT2 cells. This indicates AT2 cells also signal to mesenchymal niche cells to remodel their niche during regeneration (Paris et al. 2020). Notch signaling has been identified as a suppressor of AT2-to-AT1 cell differentiation. In response to alveolar epithelial injury, down-regulation of Notch signaling in AT2 cells promotes their transition into AT1 cells, thereby facilitating alveolar repair (Finn et al. 2019).

The inflammatory niche also regulates alveolar regeneration. Interstitial macrophage-derived IL-1β primes a subset of AT2 cells to promote their conversion into damage-associated transient progenitors (DATPs) via a HIF1α-mediated glycolysis pathway, which is required for mature AT1 cell differentiation (Choi et al. 2020). Importantly, chronic inflammation mediated by IL-1β prevents AT1 cell differentiation, leading to aberrant accumulation of DATPs and impaired alveolar regeneration (Choi et al. 2020). Another study found that IL-1 and TNF-α enhance the proliferation of AT2 cells. Expression of IL-1β and TNFα are induced in the AT2 cells niche following influenza-infection (Katsura et al. 2019). In addition to signal molecules, biophysical cues also contribute to AT2 cell fate decision via acting through hippo signaling pathways (Liu et al. 2016; Tang et al. 2018). Although tremendous efforts have been made to understand the complexity of alveolar niche in past decades, the cell composition and their dynamics during injury repair and physiological conditions, such as disease and ageing, are still not fully understood.

## Mitochondrial metabolism directs lung progenitor cells fate decision

**Fig. 3:**
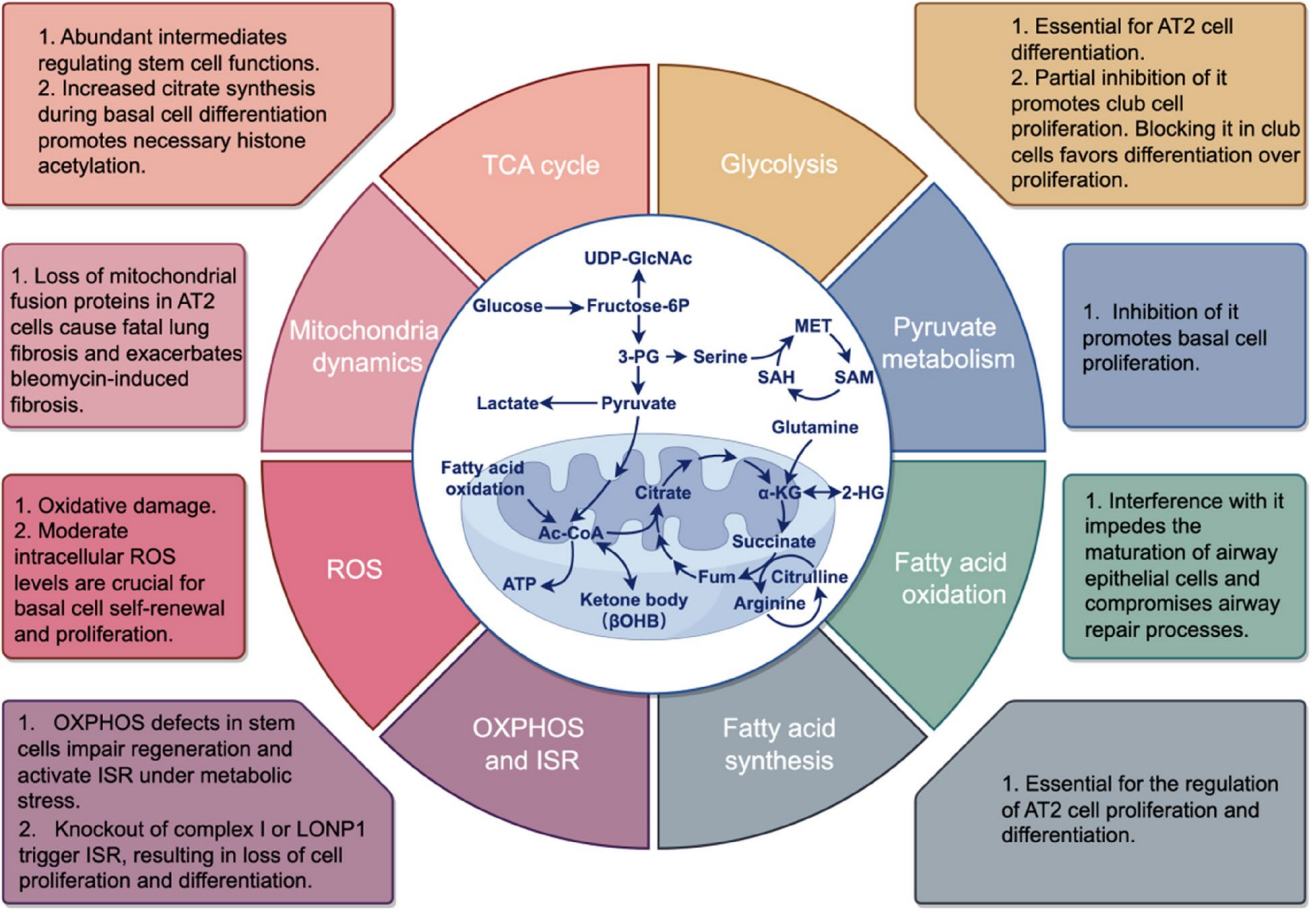
Metabolic control of lung progenitor cells fate. Cell metabolism regulates the behavior of lung progenitor cells through various metabolic pathways. This scheme outlines eight hallmarks of metabolic regulation affecting lung progenitor cell fate in this work: glycolysis, pyruvate metabolism, fatty acid oxidation (FAO), fatty acid synthesis, mitochondrial oxidative phosphorylation (OXPHOS), integrated stress response (ISR), reactive oxygen species (ROS), mitochondrial dynamics, and the tricarboxylic acid (TCA) cycle

Cellular metabolism is a complex biochemical process through which cells manufacture materials and energy. However, the impact of metabolic pathways, beyond energetic support, on the maintenance and differentiation of lung progenitor cells remains unclear. Increasing evidence suggests that cell metabolic state is critical for cell fate decision, as it can affect various aspects of cellular activity, such as signal transduction, epigenetic regulation, intracellular pH, and redox balance (Fig. 3). These discoveries change the traditional view of metabolism as simply providing energy for cellular processes. Rather, it is actively involved in regulating cell function. Here are some key points highlighting how metabolism regulates lung progenitor cell fate.

### Metabolic pathways

#### Glycolysis

Glycolysis is a fundamental metabolic pathway occurring in the cytoplasm of cells, wherein glucose undergoes ten enzyme-catalyzed reactions to produce energy. A recent study found that during alveolar regeneration, activated AMPK-PFKFB2 signaling upregulates glycolysis, which supports the intracellular energy required for cytoskeletal remodeling during AT2 cell differentiation (Wang et al. 2023). Interestingly, activating AMPK-PFKFB2 signaling in AT2 cells can rescue defective alveolar regeneration in aged mice (Wang et al. 2023). Another study found that genetic knockdown of *glucose transporter 1* (*Glut1*), selective inhibition of glycolytic metabolic enzymes, or blockade of the pentose phosphate pathway impaired AT2 cell proliferation in vitro (Li et al. 2020).

Glycolysis also regulates the function of airway progenitor cells. Reducing the availability of glucose or partially inhibiting glycolysis promote the proliferative ability of v-club progenitors and their progeny club cells, but a complete lack of glucose or blocking the glycolysis of v-club cells eliminates their increased proliferative ability. In contrast, it promotes ciliated and goblet cell differentiation (Li et al. 2019). We recently reported that pyruvate metabolism regulates basal cell fate decision. Inhibition of the mitochondrial pyruvate metabolism via disrupting mitochondrial pyruvate carrier promotes basal cell proliferation at the expense of differentiation (Li et al. 2024a, b).

#### Fatty acid metabolism

Fatty acid oxidation (FAO) is the process of metabolizing long-chain fatty acids into acetyl-CoA and further metabolizing them in the tricarboxylic acid cycle (TCA cycle), which primarily proceeds in the mitochondrial matrix. Although some tissues preferentially use fatty acids to produce energy, such as the heart, lung tissue does not rely primarily on FAO for energy under normal circumstances (Fisher 1984; Fisher et al. 1974). While β-oxidation can generate significant amount of ATP, compared to glycolysis, it requires a greater number of steps, which leads to relatively slower efficiency (Olpin 2013). Moreover, excessive reliance on FAO might lead to inefficient oxygen utilization (Panov et al. 2022), potential severe oxidative stress (Chen et al. 2019; Panov et al. 2022), mitochondrial overload induced acetyl-CoA accumulation, and metabolic imbalance (Olpin 2013). Recent research demonstrated a correlation between basal cell differentiation and the shift in cellular metabolism from glycolysis to fatty acid β-oxidation. Interference with fatty acid β-oxidation through pharmacological or genetic approaches impedes the maturation of airway epithelial cells and compromises airway repair processes. Mechanistically, fatty acid β-oxidation links to the hexosamine biosynthesis pathway to support protein glycosylation in airway epithelial cells (Crotta et al. 2023).

In addition to FAO, studies also indicate a role of fatty acid synthesis in regulating lung progenitor cell function. One important function of AT2 cells is synthesis of alveolar surfactants, which is crucial for normal lung function (Harayama et al. 2014). During regeneration, autophagy acts as a switch, leading to downregulation of lipid metabolism and upregulation of glucose metabolism in AT2 cells in response to lung injury. (Li et al. 2020). Chemical blocking of ATP citrate lyase, which converts citrate into acetyl-CoA for fatty acid synthesis and protein modification, promotes AT2 cell organoid growth (Li et al. 2020). In conclusion, fatty acid synthesis appears to negatively regulate AT2 cell differentiation. However, the in vivo role of lipid metabolism in controlling lung progenitor cell fate decision is still unknown.

#### Amino acid metabolism

Amino acids are the building blocks for protein synthesis. Additionally, they can also be used as substrates for biosynthetic reactions. Recent single-cell sequencing analyses of IPF lungs and fibrotic mouse lungs induced by bleomycin revealed a significant downregulation of enzymes responsible for glutamine metabolism, such as glutaminase-1 (*Gls1*) and glutamate-pyruvate transaminase-2 (*Gp2*) in AT2 cells (Wang et al. 2022). Further analysis suggests that the downregulation of these enzymes results in reduced proliferation and differentiation of AT2 cells in vitro (Wang et al. 2022). Current understanding of amino acid metabolism in regulating lung progenitor cell function remains limited.

### Mitochondrial oxidative stress

#### Mitochondrial oxidative phosphorylation (OXPHOS) and integrated stress response (ISR)

Mitochondrial oxidative phosphorylation (OXPHOS) serves as an important pathway for ATP production in all cells. This essential biochemical process is supported by five enzyme complexes (complexes I-V) that constitute the electron transport chain (ETC). Defects in OXPHOS, which potentially impair the self-renewal and regenerative capabilities of progenitor cells following injury, have been observed in various tissues. Such defects may activate the integrated stress response (ISR) which inhibits protein synthesis globally but enhances the translation of selective genes as an adaptive mechanism to counter metabolic stress (Han et al. 2023).

Inactivation of complex I in mouse lung epithelial cells results in postnatal lethality. Affected lungs exhibit increased transitional cells with characteristics of both AT2 and AT1 cells, along with high ISR response. Treatment with ISR inhibitors or NAD^+^ precursors partially rescue the lethality caused by Complex I dysfunction (Han et al. 2023). Interestingly, loss of complex II does not lead to ISR activation or lethality (Han et al. 2023). A recent study reported that inactivation of *Lonp1*, a mitochondrial ATP-dependent protease, in the mouse lung epithelium also led to ISR activation and dysfunction of lung epithelial cells (Xu et al. 2024).

#### Reactive oxygen species (ROS)

Reactive oxygen species (ROS), traditionally viewed as purely detrimental, have recently been discovered to work as key regulator to control progenitor cell function (Wang et al. 2013). In mouse and human basal cells, moderate ROS is essential for stem cell capacity. Alterations in ROS level activate *Nrf2*, which in turn stimulates the Notch pathway to enhance basal cell self-renewal and triggers an antioxidant program to return ROS level to a low state to maintain homeostasis balance (Paul et al. 2014). Glutathione peroxidase 4 (GPX4) is an enzyme that belongs to the family of glutathione peroxidases, which are crucial for protecting cells from oxidative damage by reducing hydrogen peroxide, organic hydroperoxides, and lipid hydroperoxides (Li et al. 2022a, b). During lung injuries, such as those caused by infections, lipopolysaccharide (LPS), or ischemia–reperfusion, *Gpx4* expression is often downregulated, leading to increased lipid peroxidation and lung tissue damage, and upregulation of *Gpx4* or the use of ferroptosis inhibitors can alleviate lung injury (Li et al. 2022a, b). Thus, while ROS modulation is critical for progenitor cell function and repair processes, maintaining appropriate levels of GPX4 activity is equally essential for protecting against oxidative damage and preserving lung tissue integrity.

### Mitochondrial dynamics

Mitochondria are highly dynamic organelles that continuously change their morphology through opposing membrane fission and fusion processes (Friedman et al. 2014). Mitochondrial fission results in small, round mitochondria, while fusion leads to elongated, interconnected mitochondrial networks. These morphological changes directly impact mitochondrial function, which also affect progenitor cell behavior and function (Ren et al. 2020). Previous studies have indicated that the absence of mitochondrial fusion proteins mitofusin 1 (*Mfn1*) and mitofusin 2 (*Mfn2*) in murine AT2 cells results in spontaneous lung fibrosis (Chung et al. 2019). Furthermore, long-term cigarette smoke exposure significantly increased the expression of specific fission and fusion marker genes, such as *Fis1, Mfn1, Mfn2, Drp1,* and *Opa1* (Hoffmann et al. 2013).

### Tricarboxylic acid (TCA) cycle

Tricarboxylic acid (TCA) cycle, also called the citric acid cycle or the Krebs cycle, constitutes a closed-loop sequence of reactions that serves as a metabolic powerhouse within cells. The TCA cycle not only supports energy metabolism, but also regulates cell functions through its intermediates (Martinez-Reyes et al. 2020). TCA cycle metabolites, such as acetyl-CoA (Lee et al. 2014a, b, c; Moussaieff et al. 2015), alpha-ketoglutarate (Carey et al. 2015; Kaelin et al. 2008; Liu et al. 2017; Tennant et al. 2009), and succinate (Letouze et al. 2013), can act as substrates or co-factors of many chromatin-modifying enzymes thereby regulating gene expression via epigenetic modifications. Currently, our knowledge of how TCA cycle activity affects lung progenitor cell function remains limited. Our group recently reported that pyruvate-mediated citrate synthesis promotes histone acetylation, which is required for basal cell differentiation (Li et al. 2024a, b).

## Dysfunction of progenitors in lung disease and progenitor cell-based cell therapy

Lung diseases remain a devastating cause of morbidity and mortality worldwide. Recent advances in lung biology indicate that progenitor cell dysfunction is a major cause of lung disease. Using progenitor cells as drugs shows promising for the treatment of lung disease.

### Dysregulated progenitor cell function in lung diseases

#### Pulmonary Fibrosis (PF)

Pulmonary fibrosis (PF) is a deadly disease characterized by the formation of scar tissue in the lungs, which impairs normal respiratory function. There are more than 200 different types of PF, with idiopathic pulmonary fibrosis (IPF) being the most common, and the exact cause remains unknown (Kamiya et al. 2024; Schneider et al. 2021). The incidence of fibrotic lung diseases such as IPF is on the rise due to the unprecedented aging of the world population (Raghu et al. 2016). Several factors have been identified to contribute to lung fibrosis, include autoimmune diseases, environmental exposures like asbestos and silica, certain medications, radiation therapy, viral infections, gastroesophageal reflux disease, and aging.

The prevailing model explaining IPF pathogenesis has centered on epithelial injury and abnormal wound repair. Recurrent microinjuries in vulnerable alveolar epithelium drive fibrotic remodeling (Lederer et al. 2018). An increasing number of new studies suggest that dysregulation of AT2 cell differentiation is a major cause of lung fibrosis. In IPF lungs, it has been found that a subset of AT2s enter and persist in an aberrant transitional state. These cells can induce fibrosis via activating myofibroblast differentiation by profibrotic secretome (Hoffman et al. 2024; Wu et al. 2020; Zhao et al. 2024). Dysfunction in AT2 cells quality control mechanisms, including the ubiquitin–proteasome system, unfolded protein response, autophagy, mitophagy, and telomere maintenance, leads to various cellular and molecular abnormalities. These include endoplasmic reticulum stress, impaired autophagy, mitochondrial dysfunction, apoptosis, inflammation, profibrotic signaling, which may eventually lead to AT2 cells dysfunction (Katzen et al. 2020).

Several lines of evidence indicate that dysregulation of lipid, glucose, and amino acid metabolism in AT2 cells contribute to the development of PF (Li et al. 2022a, b). In IPF lungs, the AT2 cells exhibit significant changes in the expression of lipid metabolism-related genes, primarily associated with the cytidine diphosphate diacylglycerol pathway, cholesterol metabolism, and triglyceride synthesis (Shi et al. 2024). Changes in fatty acid composition lead to endoplasmic reticulum stress and more ROS production in AT2 cells. This results in abnormal lipid uptake on the lung surface and reduced lipid production due to mitochondrial damage. During bleomycin injury, increased expression of glycolysis-related genes and rate-controlling genes of pentose phosphate pathway, such as phosphoglycerate mutase (*Pgam*), enolase 1 (*Eno1*), aldolase A fructose-bisphosphate (*Aldoa*), and glucose-6-phosphate dehydrogenase X-linked (*G6pdx*), has been reported (Li et al. 2020). In addition, increased dimethylarginine dimethylaminohydrolase (DDAH) activity inhibits the nitric oxide synthase (NOS) inhibitor asymmetric dimethylarginine (ADMA), expanding the pool of fibroblasts (Li et al. 2022a, b). A preprint study using murine and human iPSC-derived AT2 cell models carrying SP-CI73T, a mutation identified in human patients, found that AMPK agonists (such as AICAR and Metformin) alleviate the metabolic abnormalities of these AT2 cells, thus successfully alleviating lung fibrosis in vivo (Rodríguez et al. 2024).

Pirfenidone and nintedanib are currently used drugs for IPF treatment. They have proven to be effective in managing IPF by slowing disease progression. However, it is important to note that these drugs do not reverse existing lung damage or cure IPF (Amati et al. 2023; Chianese et al. 2024; Man et al. 2024). Completely preventing fibrosis progression remains unresolved, primarily due to our limited understanding of its underlying mechanisms. While the treatment of IPF is challenging, potential approaches, including deactivating overactive fibroblasts, enhancing collagen degradation, employing progenitor cell therapies and tissue engineering, may provide new therapeutic treatment for this disease.

#### Chronic Obstructive Pulmonary Disease (COPD)

Chronic Obstructive Pulmonary Disease (COPD) is a progressive lung disease characterized by persistent airflow limitation and breathing difficulties (Li et al. 2024a, b). It encompasses two main conditions: chronic bronchitis and emphysema. Emphysema usually refers to destruction of the tiny air sacs at the end of the airways in the lungs. Chronic bronchitis refers to a chronic cough with the production of phlegm resulting from inflammation in the airways.

Smoking and air pollution are the most common causes of COPD, which can lead to changes in lung structure, excessive mucus secretion, inflammation, and swelling of the airway lining (Antunes et al. 2021). COPD is more prevalent in the elders. The age-related changes may be abnormally augmented in COPD patients due to several factors, such as telomere shortening, cellular senescence, activation of the phosphatidylinositol-3-kinase (PI3K)/mammalian target of rapamycin (mTOR) signaling pathway, defective DNA repair, abnormal microRNA patterns, epigenetic alterations, decreased anti-aging molecules, mitochondrial dysfunction, impaired autophagy, senescence, and progenitor cell exhaustion (Barnes 2017; Hikichi et al. 2019). Moreover, impaired alveolar epithelial repair in the context of COPD has been linked to diminished Wnt signaling within the disease microenvironment, which might be caused by a transition from canonical to noncanonical Wnt signaling pathways triggered by harmful factors like cigarette smoke (Baarsma et al. 2017; Heijink et al. 2013). The Sonic hedgehog (*Shh*) signaling pathway regulates communication between mesenchymal and epithelial cells in the adult lung, primarily through the interaction of SHH with its receptor Patched, which activates the Smoothened protein to transduce intracellular signals. Abnormal activation of Shh signaling is associated with emphysema, which promotes the expansion of a specific fibroblast population that supports the proliferation of tissue-resident lymphocytes. (Wang et al. 2023).

Metabolic dysfunction in the epithelium has also been identified in COPD patients (Cloonan et al. 2016). Cigarette smoke, the primary cause of COPD, reduces mitochondrial OXPHOS in lung epithelial cells (Mizumura et al. 2014; van der Toorn et al. 2007). Moreover, smoking leads to increased fatty acid oxidation activity in AT2 cells (Agarwal et al. 2014). Furthermore, key metabolic intermediates, such as acetyl-CoA and succinate are reduced in the basal cells of smokers (Deeb et al. 2016). It has been shown that the mitochondria in patients with COPD exhibits swelling structure and impaired cristae integrity (Hara et al. 2013; Hoffmann et al. 2013). The susceptibility of mitochondrial DNA to oxidative damage may be the cause of mitochondria dysfunction (Kosmider et al. 2019). Supplementing with exogenous citrate can partially rescue the impaired differentiation of basal cell isolated from COPD patients (Li et al. 2024a, b). Further research is required to fully elucidate the mechanisms of COPD pathogenesis and to develop effective therapies for this lung disease.

### Lung progenitor cell-based therapy

Engraftment of exogenous cells in the lung for structural repair and regeneration is an area of active research but remains challenging and controversial. Early studies in the 2000s suggested that bone marrow-derived cells and other stem/progenitor cell populations could engraft and differentiate into lung epithelial cells (Weiss et al. 2011, 2008). Although many initial reports had methodological issues that led to misinterpretations, recent studies show that engrafting lung progenitor cells for improved repair and regeneration is still feasible. Here, we listed clinical trials and relevant reports related to lung stem cell therapies (Table 1). For example, lung epithelial progenitor cells isolated at embryonic day 15.5 (E15.5) showed the highest engraftment potential compared to cells isolated at other developmental stages. While engraftment has been demonstrated in animal models, the reconstruction of damaged alveolar structures is limited. Engrafted cells may repopulate existing lung structures rather than rebuilding new ones (Rosen et al. 2015; Shiraishi et al. 2019). Recently, pluripotent stem cell (PSC)-derived lung epithelial cells show promise for their potential for transplantation. These cells can integrate into bleomycin-injured lungs of immunocompetent mice (Herriges et al. 2023; Ma et al. 2023). Specifically, engrafted cells can persist for at least six months and produce functional AT2 and AT1 cells (Herriges et al. 2023, Ma et al. 2023, Milman Krentsis et al. 2024). Recent studies have also shown that transplantation of p63^+^ progenitor cells into the lungs of bleomycin-injured mice and COPD mice promotes lung function recovery (Wang et al. 2021). A clinical trial involving patients with stage II to IV COPD revealed that transplantation of autologous p63^+^ progenitor cells in patients from the intervention group led to an average increase of 18.2% in gas transfer capacity in 24 weeks after transplantation, whereas the control group experienced a 17.4% decline in gas transfer capacity. Moreover, participants in the intervention group showed increased walking distance by more than 30 m within six minutes. Comparison of the transcriptome analysis of precursor cells between patients responded differently from the treatment indicates that high expression of P63 correlates with the treatment effect (Wang et al. 2024). However, despite advancements, significant obstacles remain before the clinical implementation of cell therapies for lung regeneration. These challenges include optimizing delivery methods, ensuring the long-term survival of engrafted cells, and addressing potential safety concerns.

**Table 1:** Clinical trials and relevant reports related to lung stem cell therapies

Abstract	Cell Description	Reference
Intravenously administered murine canalicular-stage fetal lung progenitors can be successfully engrafted into irradiated mice with naphthalene-induced damage to the airway	Fetal human and mouse lungs harvested at the canalicular phase of lung development	Rosen et al. 2015
Embryonic day 15.5 (E15.5) lung epithelial cells show the highest engraftment potential. Preconditioning of the recipient lung appears to be important for successful engraftment	E15.5 mouse whole lung cells	Shiraishi et al. 2019
Pluripotent stem cell (PSC)-derived lung epithelial cells show promise for their potential for transplantation. These cells can integrate into bleomycin-injured lungs of immunocompetent mice. Engrafted cells can persist for at least 6 months and produce functional AT2 and AT1 cells	Mouse PSC-derived alveolar epithelial progenitor cells	Herriges et al. 2023
Transplantation of mouse or human primary (BC) or PSC-derived (iBC) basal stem cells into injured airway epithelium leads to self-renewing, multilineage-differentiating engrafted BCs. iBCs persist for over two years in syngeneic mice and can be serially transplanted through seven generations	Mouse or human BC or iBCs	Ma et al. 2023
Engraftment of donor lung progenitor cell without conditioning leads to the formation of donor-derived alveolar patches and attenuation of fibrosis	Adult mouse whole lung cells	Milman Krentsis et al. 2024
Functional lung structure could be reconstituted by intrapulmonary transplantation of p63^+^ progenitor cells	Mouse and human p63^+^ progenitor cells	Wang et al. 2021
Transplantation of autologous P63^+^ precursor cells improve the lung function of patients with stage II to IV COPD	p63^+^ progenitor cells	Wang et al. 2024

## Conclusions and perspectives

In conclusion, our review highlights the growing understanding of lung progenitor cell biology and their pivotal roles in lung homeostasis and disease. This comprehensive overview lays the groundwork for leveraging our knowledge of lung progenitor cells to advance therapeutic strategies for lung diseases.

However, despite centuries of research into lung diseases, some deadly lung diseases, such as IPF, remain incurable. A key limitation is that nearly all current research is conducted on mouse models. While human and mouse lungs are generally similar, differences in their structure and cell compositions still exist. Future studies could benefit from incorporating additional animal models, such as ferrets and primates, to address this gap. Another challenge is that existing disease models do not fully recapitulate the pathological conditions observed in humans. For example, while bleomycin administration is the prevalent method for inducing lung fibrosis, they are significantly distinct from the fibrotic patterns and irreversibility seen in IPF patients. For models using genetically deficient mice, inducing genetic defects in all cells of the same type simultaneously does not accurately reflect the disease conditions. Therefore, to better understand and address the causes of these life threatened lung diseases, there is an urgent need for the development of more representative models. Lastly, our understanding of how aging affects lung function remains limited. It is known that the incidence and severity of many lung diseases are strongly age-related. For instance, IPF commonly occurs between the ages of 50 and 70, while non-specific interstitial pneumonia typically manifests between 50 and 60 years of age. Similarly, the difference of disease prevalence between genders is also a less studied direction in the field. Collaborations across disciplines and the connection of fundamental research to clinical practice are essential for capitalizing on these insights and tackling the complex challenges posed by lung diseases.

## Data Availability

Not applicable.

## Abbreviations

AGER: Advanced glycosylation end-product specific receptor
AT1 cells: Alveolar type 1 cells
AT2 cells: Alveolar type 2 cells
BASCs: Bronchoalveolar stem cells
CCSP: Club cell secretory protein
CLDN4: Claudin 4
COPD: Chronic obstructive pulmonary disease
CYP2F2: Cytochrome P450, family 2, subfamily f, polypeptide 2
DATPs: Damage-associated transient progenitors
DASCs: Distal airway stem cells
DSG3: Desmoglein 3
ECs: Endothelial cells
ECM: Extracellular matrix
ETC: Electron transport chain
FAO: Fatty acid oxidation
GPX4: Glutathione peroxidase 4
ICZ: Intercartilaginous zone
IPF: Idiopathic pulmonary fibrosis
iPSCs: Induced pluripotent stem cells
ISR: Integrated stress response
KRT5: Keratin 5
KRT6a: Keratin 6A
KRT8: Keratin 8
KRT13: Keratin 13
KRT14: Keratin 14
LNEPs: Lineage-negative epithelial progenitors
LPS: Lipopolysaccharide
MANCs: Mesenchymal alveolar niche cells
MFN1: Mitochondrial fusion proteins mitofusin 1
MFN2: Mitofusin 2
MYH11: Myosin heavy chain 11
NGFR: Nerve growth factor receptor
OXPHOS: Mitochondrial oxidative phosphorylation
PDPN: Podoplanin
PF: Pulmonary fibrosis
PNECs: Pulmonary neuroendocrine cells
ROS: Reactive oxygen species
SCGB1A1: Secretoglobin family 1A member 1
SCGB3A2: Secretoglobin family 3A member 2
SMGs: Submucosal glands
SHH: Sonic Hedgehog
TCA cycle: Tricarboxylic acid cycle
P63: Transformation related protein 63
UPK3A: Uroplakin 3A
V-club cells: Variant-club cells
2OGDD: 2-Oxoglutarate-dependent dioxygenases
